# Three-layered semantic framework for public health intelligence

**DOI:** 10.1186/s13326-025-00338-1

**Published:** 2025-09-15

**Authors:** Sathvik Guru Rao, Pranitha Rokkam, Bide Zhang, Astghik Sargsyan, Abish Kaladharan, Priya Sethumadhavan, Marc Jacobs, Martin Hofmann-Apitius, Alpha Tom Kodamullil

**Affiliations:** 1 Department of Bioinformatics, Fraunhofer Institute for Algorithms and Scientific Computing (SCAI), Schloss Birlinghoven, Sankt Augustin, 53757 Germany; 2 Bonn-Aachen International Center for Information Technology (B-IT), University of Bonn, Bonn, 53113 Germany; 3 University of Bonn, Bonn, 53113 Germany; 4 Causality Biomodels, Kinfra Hi-Tech Park, Kalamassery, Cochin, 683503 Kerala India

**Keywords:** Semantic framework, Ontology, Data interoperability, Data integration, Semantic web, Web of data, Public health intelligence

## Abstract

**Background:**

Disease surveillance systems play a crucial role in monitoring and preventing infectious diseases. However, the current landscape, primarily focused on fragmented health data, poses challenges to contextual understanding and decision-making. This paper addresses this issue by proposing a semantic framework using ontologies to provide a unified data representation for seamless integration. The paper demonstrates the effectiveness of this approach using a case study of a COVID-19 incident at a football game in Italy.

**Method:**

In this study, we undertook a comprehensive approach to gather and analyze data for the development of ontologies within the realm of pandemic intelligence. Multiple ontologies were meticulously crafted to cater to different domains related to pandemic intelligence, such as healthcare systems, mass gatherings, travel, and diseases. The ontologies were classified into top-level, domain, and application layers. This classification facilitated the development of a three-layered architecture, promoting reusability, and consistency in knowledge representation, and serving as the backbone of our semantic framework.

**Result:**

Through the utilization of our semantic framework, we accomplished semantic enrichment of both structured and unstructured data. The integration of data from diverse sources involved mapping to ontology concepts, leading to the creation and storage of RDF triples in the triple store. This process resulted in the construction of linked data, ultimately enhancing the discoverability and accessibility of valuable insights. Furthermore, our anomaly detection algorithm effectively leveraged knowledge graphs extracted from the triple store, employing semantic relationships to discern patterns and anomalies within the data. Notably, this capability was exemplified by the identification of correlations between a football game and a COVID-19 event occurring at the same location and time.

**Conclusion:**

The framework showcased its capability to address intricate, multi-domain queries and support diverse levels of detail. Additionally, it demonstrated proficiency in data analysis and visualization, generating graphs that depict patterns and trends; however, challenges related to ontology maintenance, alignment, and mapping must be addressed for the approach’s optimal utilization.

## Background

We have witnessed the emergence of infectious diseases leading to global pandemics, posing a substantial threat to individuals and the global community. In the year 2020 alone, the world witnessed the World Health Organization (WHO) responding to 116 crises across 194 locations worldwide [1]. This accentuates the severity of the difficulty we encounter in managing and lessening the impact of health crises like the one being discussed. The ability to predict and estimate the burden on healthcare systems beforehand is crucial, and this is where surveillance systems come into play. Surveillance, in the context of public health, involves the continuous, systematic collection, analysis, and interpretation of data [2]. This data-driven process is the backbone for health planning, implementing interventions, and assessing policies and practices.

Epidemiologists have long relied on epidemic intelligence tools to respond effectively to outbreaks. The World Health Organization (WHO) established the Global Outbreak Alert and Response Network (GOARN) [3] in 2000, aiming to consolidate efforts among technical institutions, research bodies, universities, and international health organizations to bolster global preparedness and response to disease outbreaks.

Health informatics has revolutionized disease surveillance by developing electronic tools that facilitate data collection, analysis, and dissemination. However, traditional surveillance systems often provide limited contextual insights, hindering effective decision-making. Moreover, the varying analytical capabilities among stakeholders can further complicate policymakers’ processes.For instance, MedIsys [4], introduced in 2004, operates as an automatic public health surveillance system. It comprehensively monitors diverse threats, including infectious diseases, bioterrorism, and chemical, biological, radiological, and nuclear hazards, by aggregating articles from open sources and news media. Similarly, The Global Public Health Intelligence Network (GPHIN) [5] stands as a continuous event-based surveillance system, meticulously gathering and disseminating preliminary reports of public health events through monitoring global media sources. These systems are similar to the EIOS data source used in our use case.

In situations necessitating rapid response, the Early Warning, Alert and Response System (EWARS) [6] plays a pivotal role. Established with a specific focus on disease outbreaks in emergency settings, quick detection and intervention. Concurrently, the Global Infectious Disease and Epidemiology Network (GIDEON) [7] offers a comprehensive suite of tools for diagnosing, treating, and tracking infectious diseases on a global scale.

The usage of ontologies for pandemics and infectious diseases is not new. Bayoudhi et al. [8] has given an overview of the ontologies developed in this context. For instance, the infectious Disease Ontology (IDO) developed by Cowell et al. [9] has been used widely in the biomedical field and has been one of the popular ontologies used for extension into domain-specific. IDOMAL ontology [10] for malaria disease, IDOBRU ontology [11] for Brucellosis, IDODEN ontology [12] for Dengue fever, and IDOMEN ontology [13] for Meningitis, are all based on IDO. When we look specifically into our topic of interest, the COVID-19 pandemic, many ontologies are built upon IDO for COVID-19. He et al. [14] developed the COVID-19 ontology in 2020 to serve the purpose of data integration, sharing, and analysis, and in 2022, the authors updated the ontology, which has been used in many applications like standardization, NLP tasks, and data integration. The updated ontology has also been used to analyze different SARS-CoV variants and also in drug repurposing [15]. Sargsyan et al. [16] developed a COVID-19 ontology with a strong focus on chemical entities suited for drug repurposing.

While all these ontologies have been used for text mining purposes and data integration, Lusignan et al. [17] developed an ontology that was useful for identifying COVID-19 cases and monitoring the spread of the disease. However, limitations include the misclassification of false positive lab results and its development in a single sentinel system. The CODO ontology [18] includes concepts related to contact tracing, diagnosis, disease measure, and many other topics. This ontology was then used for COVID-19 contact tracing in risk detection systems, question-answer, and document annotation. Furthermore, the authors used the CODO ontology to build a knowledge graph with 5 million triples [19].

Building upon the well-defined structure provided by individual ontologies, semantic frameworks offer a powerful approach to healthcare surveillance. These frameworks leverage semantic technologies like ontologies to integrate and standardize data, support knowledge representation, and facilitate interoperability. Several semantic frameworks have been developed for specific applications, including Hospital Acquired Infections surveillance (HAIKU) which utilized the Healthcare-Associated Infections (HAI) ontology for assisting physicians in case detection, risk stratification and diagnosis [20] and the semantic framework to improve interoperability of malaria surveillance (SIEMA) [21], and IoT-based malaria control (SWoT) [22] are few other examples. The work of Baker et al. [23] underscores the importance of semantics in pandemic preparedness, demonstrating the potential of these technologies to facilitate rapid and effective responses to pandemic outbreaks. As healthcare surveillance becomes increasingly complex and data-driven, semantic technologies are poised to play an increasingly important role in protecting public health.

These frameworks, however, generally lack the capacity to incorporate contextual data, which is essential for a holistic understanding of health threats. Contextual data includes information from various domains such as geography, social behavior, environment, and health systems, plays a critical role in understanding and predicting disease dynamics. This need for context-aware intelligence led to the emergence of the pandemic and epidemic intelligence concept. The pandemic and epidemic intelligence concept emphasize the integration of contextual data from various sources, including geographical, social, environmental, and health-related information. This approach offers a more holistic understanding of disease risks and informs better-informed policies and decisions [24].

To address the limitations of existing epidemic intelligence systems, this paper proposes a novel semantic framework that enhances the integration of contextual information for pandemic surveillance and response. Our approach is grounded in semantic interoperability and linked data principles, allowing for more comprehensive, context-rich data representation by incorporating information from diverse sources. By uniting various platforms and integrating external knowledge, the framework supports early detection and prevention of pandemics through the application of semantic web technologies and domain-specific ontologies. This integration facilitates improved knowledge representation and decision-making, enabling more effective responses to emerging public health threats. To demonstrate its utility, we apply the framework to the COVID-19 pandemic, showcasing its potential to improve data integration, enhance coordination, and streamline epidemic response efforts.

## Methods

The COVID-19 pandemic declared as a global threat in March 2020 [25], significantly impacted Italy, where mass gatherings, including beloved football events, were identified as key contributors to disease spread. Specifically, the Champions League match between Valencia and Atalanta, held in Bergamo—one of Italy’s COVID-19 epicenters—has been highlighted in news articles as a factor in Italy’s coronavirus disaster. Quantitative analysis suggests that this football match, which occurred in February 2020, played a pivotal role in the pandemic’s escalation in March and April of the same year [26].

Considering this scenario as our use case, our semantic framework aims to address the existing challenge within traditional surveillance and intelligence systems. To facilitate the integration and sharing of data from diverse sources and formats, the framework proposes the utilization of ontologies. It comprises three layers of ontologies, each representing a distinct level of detail and abstraction. In the subsequent section, we will delve into the data sources utilized for our use case and the ontology development process and provide a detailed examination of our framework architecture.

### Data sources

Public health intelligence is the process of gathering, analyzing, and disseminating information about pandemics and their effects on public health and society. We collected data from various sources that are relevant to the spread of diseases, especially in the context of pandemics. One of the sources we used was Epidemic Intelligence from Open Sources (EIOS) [27], which is a global platform that provides articles published on the web related to various hazard types, threat types, and health-related information. Additionally, we utilized Europe’s COVID-19 line list data, collected by the World Health Organization (WHO), which encompasses comprehensive information on various aspects, including sociodemographics, diagnostics, exposure, health conditions, disease progression and outcomes, and signs and symptoms. The dataset is stored in WHO xMART^1^ data platform, which is not available publicly. Other essential data sources were flight data from FlightAware [28], which provides detailed information about flight origin, destination, duration, and frequency between various cities worldwide. This data proved instrumental in understanding potential disease transmission routes and associated risks. Furthermore, we incorporated data sources that offered insights into events, hazards, impacts, and prevention strategies. For instance, we utilized data regarding stadium and sports schedules^2^, enabling the identification of mass gathering occurrences and locations. We identified key concepts and their interrelationships relevant to public health by utilizing these diverse data sources. These concepts were categorized into distinct domains based on their contextual relevance and area of interest.

### Ontology development process

Ontologies are built using the Protégé tool [29] and the Web Ontology Language (OWL) [30] language. OWL offers a wide vocabulary to represent knowledge regarding classes, properties, and relationships. We have used RDF Schema (RDFS) annotations [31], which are annotations that provide additional information and documentation for the ontology elements. One of the advantages of ontology is the ability to reuse concepts. We have used terms from existing ontologies whenever possible, which we have found using the Ontology Lookup Service (OLS)^3^ developed by the European Bioinformatics Institute (EBI). This service allows us to search for relevant concepts from various ontologies. We have also used Ontofox^4^, a web-based tool that supports ontology reuse by importing selected terms and their annotations from source ontologies. Furthermore, we have collaborated with domain experts to create new terms as needed. Throughout the development process, we have adhered to the Open Biological and Biomedical Ontology (OBO) Foundry principles [32] to ensure the ontology’s quality, interoperability, and reusability.

### Framework architecture

**Fig. 1:**
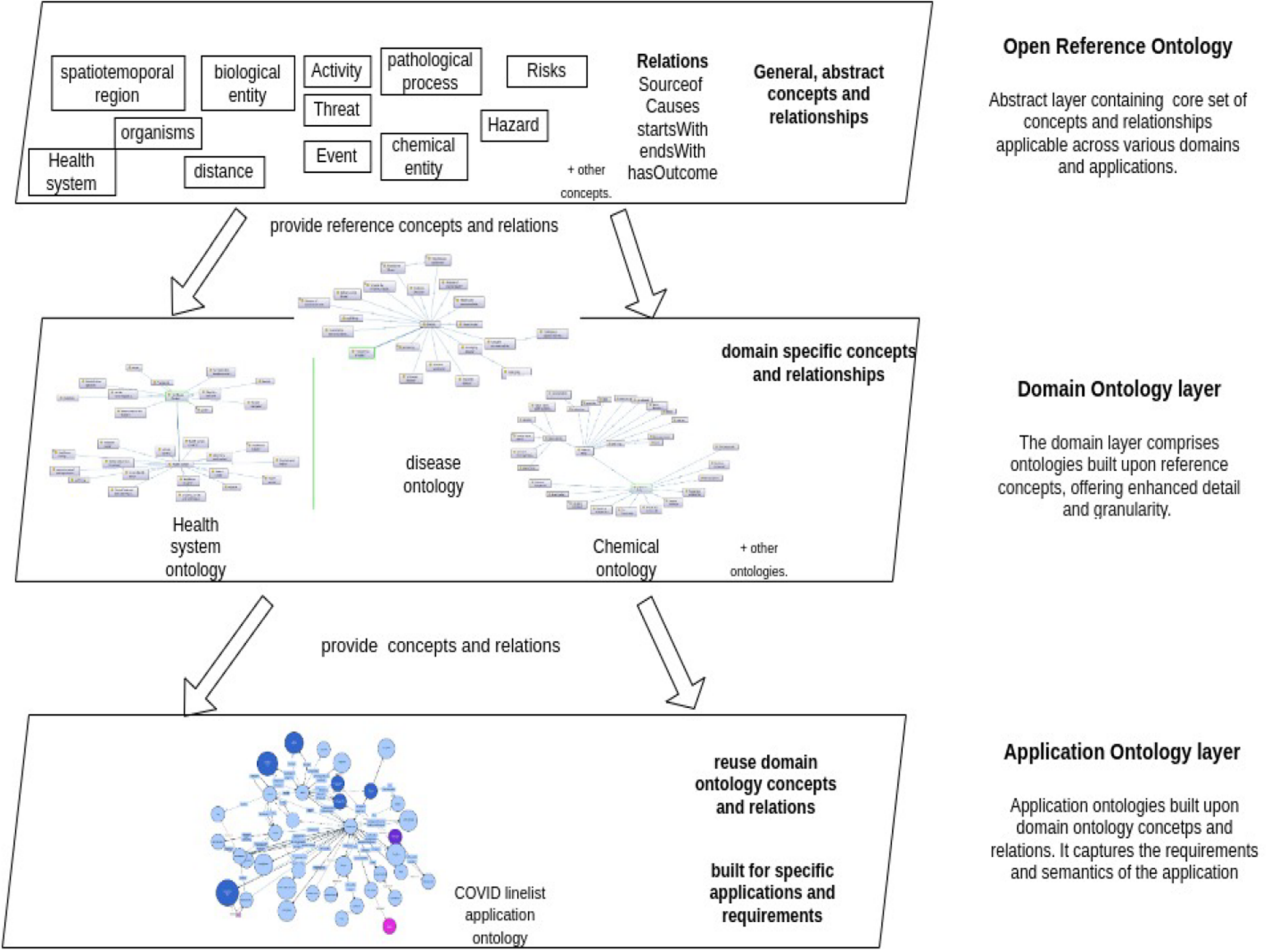
Three layered architecture

To address the complexities of public health intelligence, we developed multiple ontologies for different domains, such as healthcare systems, mass gatherings, travel, and disease. These ontologies were carefully crafted with input from experts to capture the intricate relationships between concepts and domains, including cause-and-effect interactions, mitigation strategies, and other relevant connections. N. Guarino [33] categorises ontologies based on their “level of generality” into three types. Top-level ontologies encompass fundamental concepts like spatiotemporal entities, objects, and actions, forming a foundation independent of specific domains. These ontologies are usually equipped with a rich axiomatic layer. Additionally, there are domain ontologies and application ontologies detailing entities and information within generic domains (e.g., biology or chemicals) or generic tasks (e.g., diagnosis) by specializing concepts found in top-level ontologies. The application ontology layer describes application-specific relationships and axioms that enhance the expressiveness and reasoning of the data. By leveraging these three types of ontologies, we devised a three-layered architecture as shown in Fig. 1 that promotes reusability and flexibility. This architecture enables the integration of existing ontologies, ensuring consistent knowledge representation, and serves as a robust backbone for the overall semantic framework. A detailed examination of these layers will be is presented in the subsequent section.

#### Open reference ontology

The Open Reference Ontology (ORO) serves as a foundational framework encompassing core set of concepts and relationships applicable across various domains and applications. As the abstraction layer, it fosters interoperability and facilitates the exchange of information by providing a shared reference point for knowledge representation. It captures common semantics that can be applied to lower-level ontologies. These lower-level ontologies can leverage the relationships defined in the open reference ontology to compose information through predicates and derivation rules [34].

**Fig. 2:**
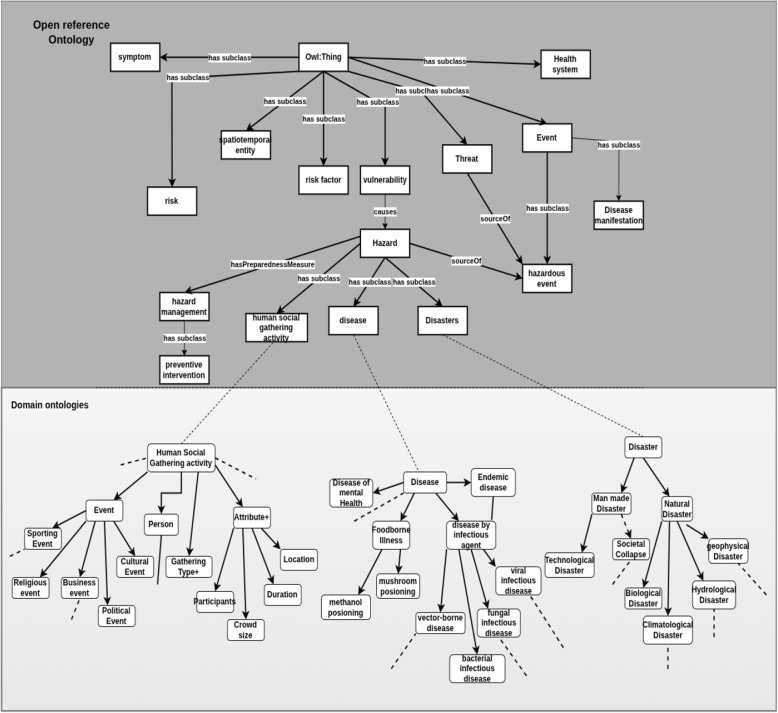
Open reference ontology

The Open Reference Ontology, illustrated in Fig. 2, was developed specifically to represent knowledge relevant to public health, threats, responses and the specific requirements of our use-case. It incorporates core concepts such as hazard, threat, *risk factor*, *vulnerability*, *disease*, *preventive measures*, *mitigation*, and impact, along with key relationships like *causes*, *effects*, *mitigates*, *prevents*, and *source of*. The ontology utilizes concepts from existing ontologies and new concept classes were created where appropriate. The Table 1 presents the ontology statistics, summarizing key metrics such as the number of classes, properties, and number of newly created concepts.

**Table 1:** Open reference ontology statistics

Class count	Object property count	Data property count	Logical Axioms count	New class & property count
99	8	0	91	20

#### Domain-specific ontologies

The Domain-specific ontologies were developed through collaboration with domain experts to extend the concepts of the open reference ontology to encompass domain-specific information and details. These ontologies cover a wide range of domains, including disease, pathogen, symptom, chemical, travel, mass gathering, and more. The selection of these domains is grounded in real-world data availability and information depth. These ontologies are invaluable when studying disease spread during mass gatherings, as they establish connections between disease-related and event-related concepts. By including concepts like population, travel routes, and preventive measures, these ontologies enable effective communication and collaboration across various disciplines by providing essential context for public health officers. A total of 16 domain ontologies were created as described in Tables 2, and 3 presents the ontology statistics like the number of classes, properties, and number of newly created concepts.

**Table 2:** Domain ontologies

Ontology	Defintion	Class count	New concepts & properties count
Chemical Ontology	Ontology comprises entities related to chemicals/molecules that can cause emergency health events/related to hazardous events	249	12
Disaster Ontology	Ontology comprises entities related to natural and man-made disasters	56	45
Diseases Ontology	Ontology comprising entities related to human and animal diseases	232	52
Foodborne Disease Ontology	Ontology comprises entities related to food, food safety, and foodborne illness	77	21
General Ontology	Ontology comprises general entities related to infectious diseases and outbreaks	38	17
health system Ontology	Ontology comprises entities related to components of a health system, such as healthcare service, healthcare workers, planning	51	15
Immunity Ontology	Ontology comprises entities related to immunity, such as types of immunity. Components of the immune system	44	2
Mass gathering	Ontology comprising of entities related to mass gathering events	166	131
Organisms Ontology	Ontology comprises entities related to organisms such as bacteria, viruses, and others, which play key roles in case of disease or outbreak	354	80
Outcomes Ontology	Ontology comprising of entities related to the outcomes of a disease or an outbreak	24	17
Population Ontology	Ontology comprises entities related to the population and its characteristics	51	11
Preventive measures	Ontology comprises entities related to steps and measures taken to prevent the spread of a disease	42	12
Surveillance Ontology	Ontology comprising of entities related to surveillance of diseases	34	39
Societal Ontology	Ontology comprising entities related to the societal impact of a disease or an outbreak	19	13
Travel Ontology	Ontology comprises entities related to travel entities or events	150	120
Zoonosis Ontology	Ontology comprises entities related to animals and their role in the transmission of infectious diseases	75	3

**Table 3:** Domain ontologies statistics

Ontology	Class count	Object property count	Data property count	Logical Axioms count
Chemical Ontology	249	0	0	256
Disaster Ontology	103	8	5	119
Disease Ontology	232	0	0	273
Foodborne Illness Ontology	77	0	0	76
General Ontology	39	0	0	40
Health System Ontology	52	0	0	50
Immunity Ontology	44	0	0	43
Mass Gathering Ontology	166	12	7	193
Organism Ontology	354	0	0	355
Outcomes Ontology	24	0	0	23
Population Ontology	51	0	0	50
Product Safety Ontology	33	0	0	63
Preventive Measures Ontology	42	0	0	41
Societal Ontology	20	0	0	37
Surveillance Ontology	35	5	18	86
Travel Ontology	150	9	8	184
Zoonosis	75	0	0	74

These domain ontologies were built by reusing concepts from existing ontologies such as NCIT (National Cancer Institute Thesaurus), CHEBI (Chemical Entities of Biological Interest), DOID (Human Disease Ontology), NCBITaxon (National Center for Biotechnology Information Taxonomy), SYMP (symptoms ontology) and HP (Human Phenotype Ontology) to provide a solid foundation for representing various concepts. These well-established ontologies ensured a comprehensive and standardized approach to defining domain-specific information. Furthermore, we incorporated geographical elements and location-related data by utilizing the Geonames ontology. This integration facilitated the effective representation and connection of location-based concepts within our ontologies.

#### Application-specific ontologies

The application ontology is built upon the concepts and relationships defined in the open reference ontology and the domain ontologies and customizes them to fit the specific requirements and functionalities of the application [33]. It also introduces new concepts that are specific to the application domain. The application ontology captures the unique aspects and semantics of the application domain by using OWL restrictions like value constraints and cardinality constraints, enabling more targeted and application-specific reasoning, as well as data integration functionality.

**Fig. 3:**
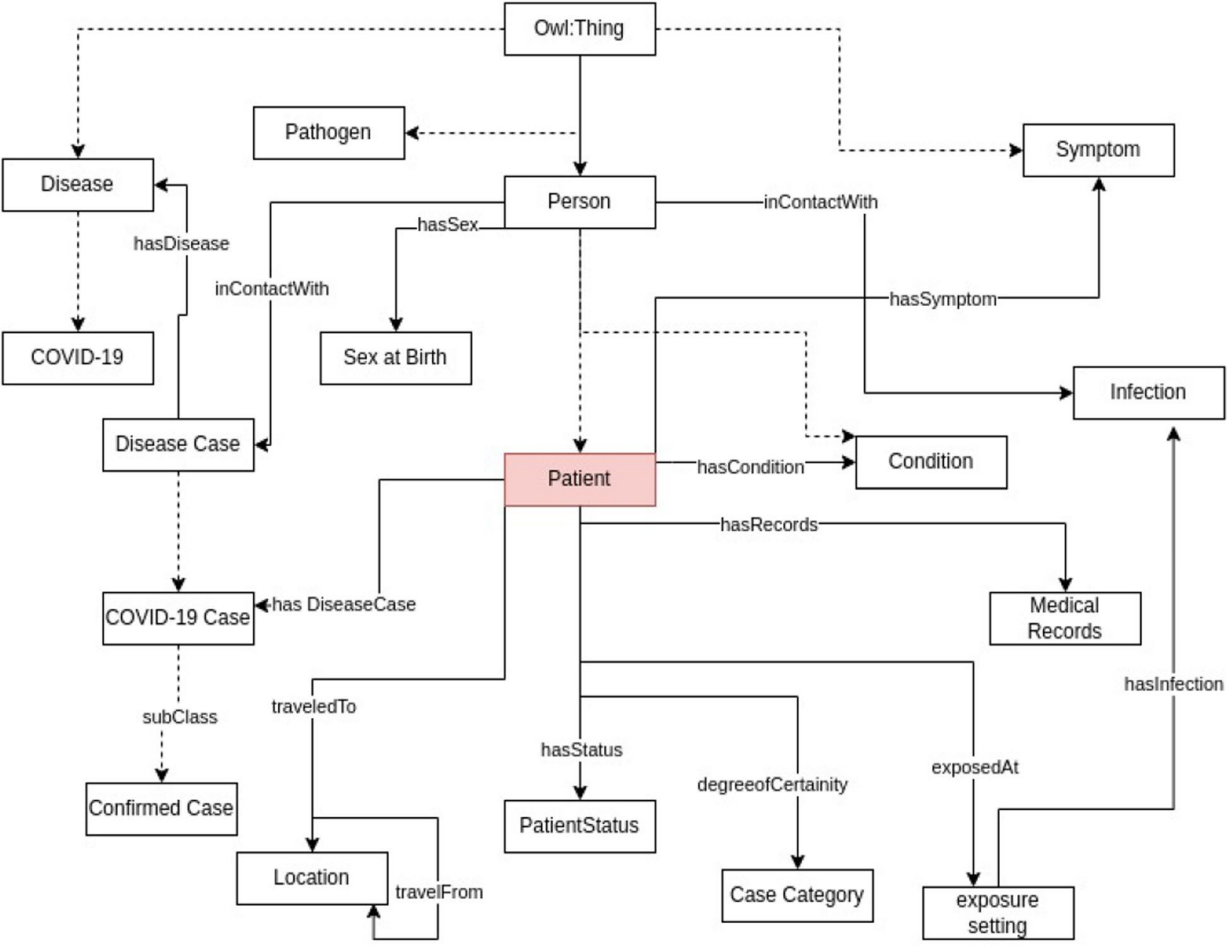
Minimalistic view of COVID-19 application ontology

For our use case, we developed an application ontology based on the COVID-19 line list dataset which contains detailed information about individual cases of COVID-19 (Fig. 3). The ontology covers concepts such as hospital information (admission date, discharge date, etc.), symptoms, comorbidities, infection exposure (dates, places, etc.), travel history of the patients, and lab test results. The ontology uses concepts from the symptoms, disease, hospital surveillance, and travel domain ontologies, as well as the spatiotemporal concepts from the open reference ontology. The ontology describes the structure and semantics of the COVID-19 line list data and enables reasoning and analysis of the data. The Table 4 presents the ontology statistics, summarizing the number of classes, properties, and number of newly created concepts.

**Table 4:** COVID-19 line list ontology (application ontology) statistics

Class count	Object property count	Data property count	Logical Axioms count	New class & property count
83	49	45	380	73

**Fig. 4:**
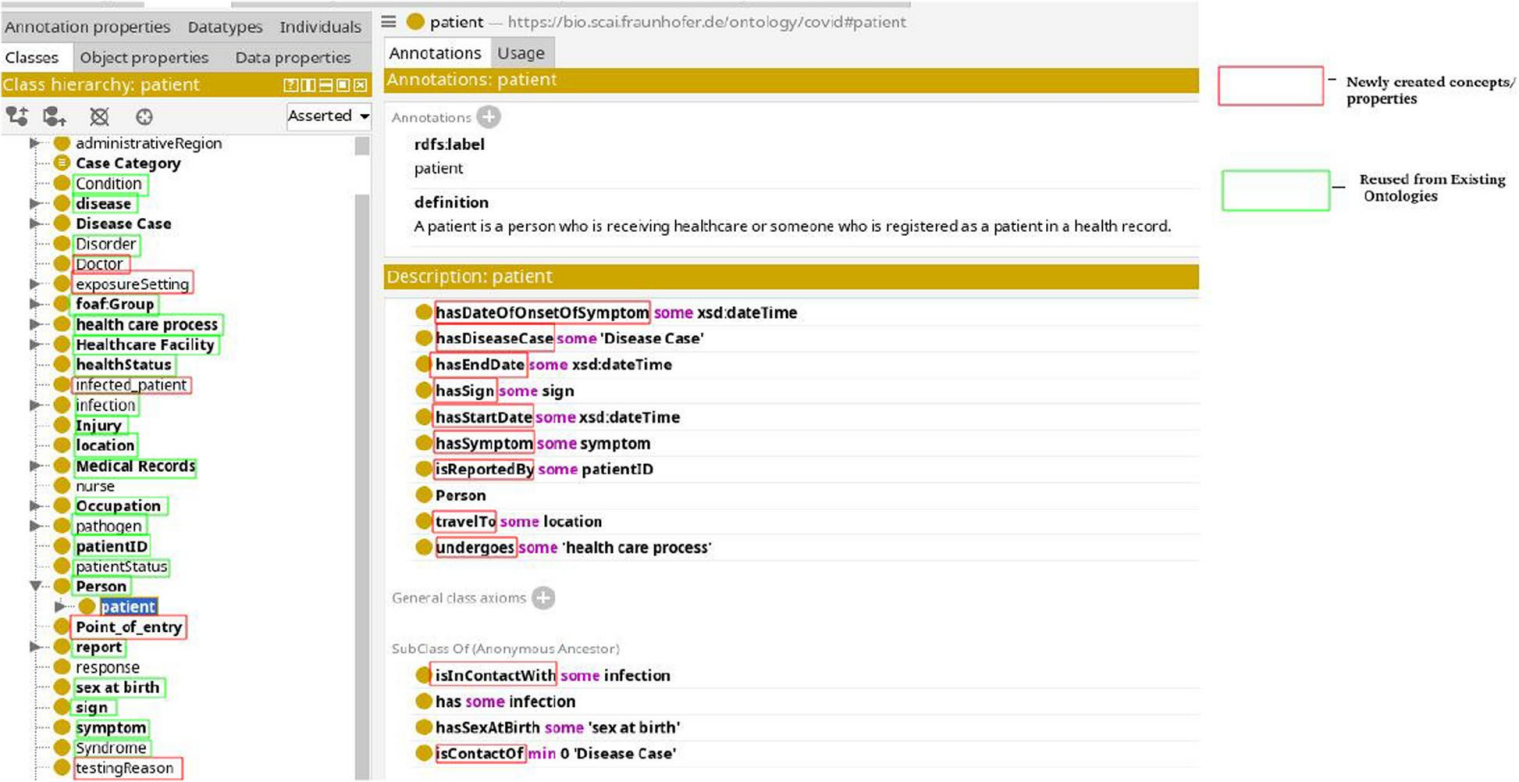
Patient class

**Fig. 5:**
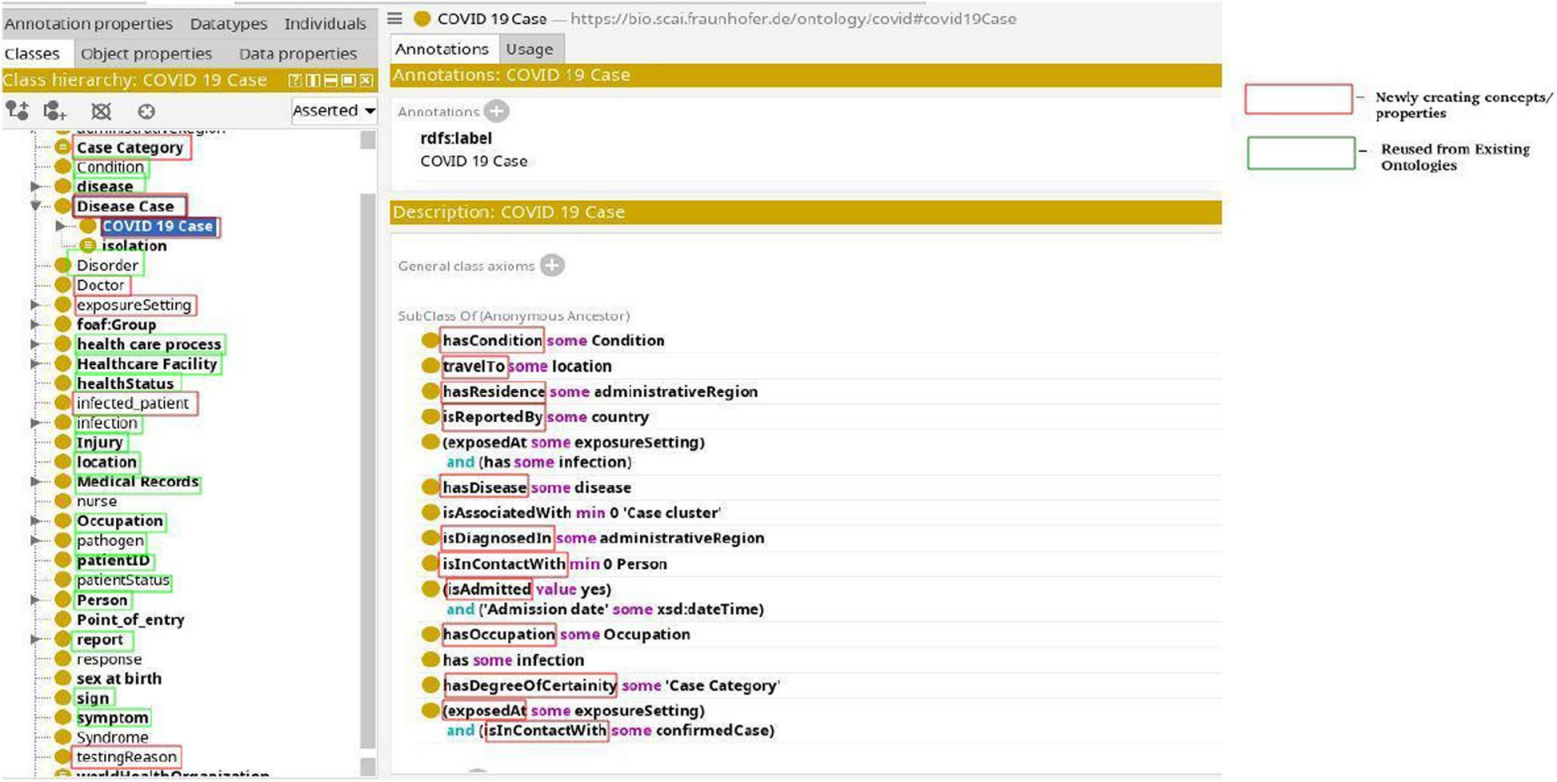
Covidcase class

Figures 4 and 5 illustrates examples of two classes, namely Patient and COVID-19 Case, respectively. These classes are interconnected through a ‘hasDiseaseCase’ relationship. The Patient instance is a subclass of the broader Person class. We’ve established a specialized class named COVID-19 Case, which inherits characteristics from both the Disease Case class and the COVID-19 disease by the ‘subClassOf’ relation. By doing so, we group disease cases specifically related to COVID-19 while also inheriting the axioms associated with Disease Case and the COVID-19 disease. This hierarchical structure streamlines the organization and analysis of data concerning COVID-19 cases. Moreover, our ontology captures critical aspects of the Patient class, featuring various axioms encompassing key attributes. For instance, we employ the travelTo relationship to connect the Patient class to locations, reflecting where a Disease Case has visited. Similarly, we employ the hasCondition relationship to characterize the health condition of Patient class. We have the hasDegreeOfCertainty relationship that connects instances of the ‘Disease Case’ category to various levels of certainty, like ‘confirmed’, ‘probable’, ‘suspect’, and discarded.

In addition to these relationships and class hierarchies, our ontology includes axioms designed to ensure data consistency and reliability. For instance, we have implemented axioms such as (isAdmitted *value* yes) to indicate whether a Disease Case has been admitted, and (‘Admission date’ *some* xsd:dateTime) to specify that if a disease case is admitted, an admission date is required. These axioms are essential checks to maintain dataset integrity and support in-depth data analysis. These are just a few examples of the relationships and classes within our ontology, designed to comprehensively model and represent the intricate web of associations and attributes pertinent to patients and disease cases.

#### System architecture and implementation

The semantic framework utilizes Apache Jena Fuseki^5^ as the triplestore and SPARQL server. RDF data, including ontologies and generated triples, are stored persistently in Jena’s TDB, a native RDF database designed for efficient storage and retrieval. Fuseki provides a SPARQL endpoint for querying and managing the data. Reasoning is performed using the RDFS Rule Reasoner^6^, which supports inference over subclass and subproperty hierarchies and enforces datatype range validation. This inference model generates implicit triples based on the ontology’s semantics, extending the explicit data. For visualization and exploration, Neo4j is employed to represent and query the knowledge graph.

## Results

In the context of our research, we harnessed the power of a semantic framework supported by ontologies to tackle the complex task of integrating and analyzing various datasets. This comprehensive approach enabled the semantic enrichment of both structured and unstructured data, resulting in a seamlessly interconnected web of data. The ontologies were used to annotate data and information and represent things. The annotation of concepts in the data sources with ontology concept URIs made them findable and accessible on the web of data.

**Fig. 6:**
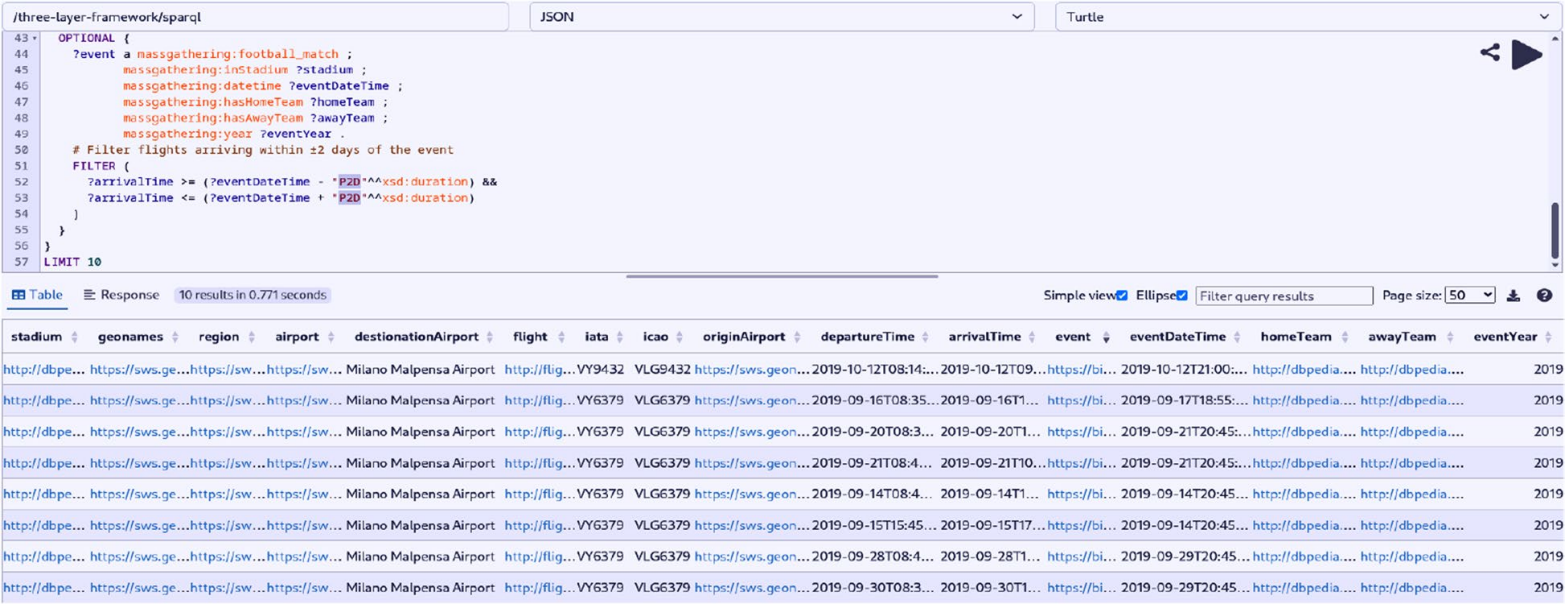
Integration of sports events, stadiums, airport and flight data

**Fig. 7:**
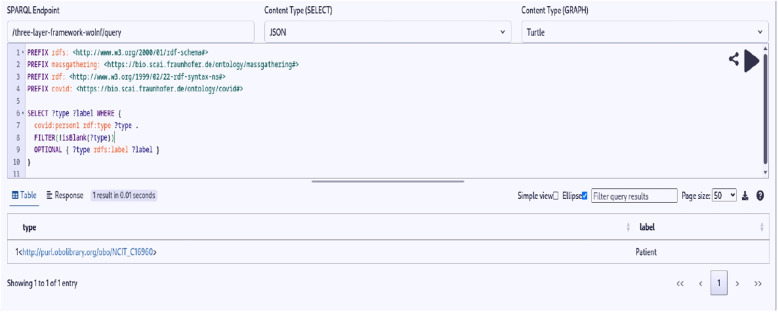
Entity *person1* type retrieval without inference

**Fig. 8:**
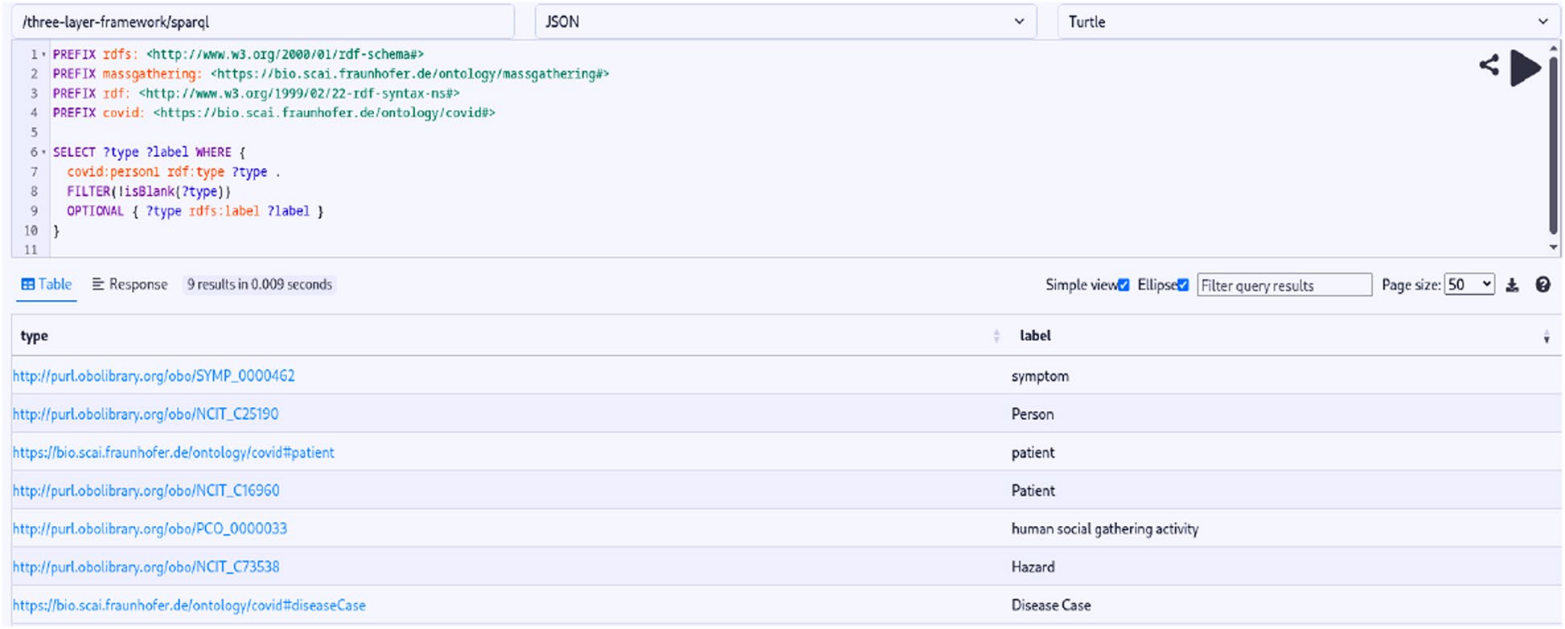
Entity *person1* type retrieval with inference model enabled

**Fig. 9:**
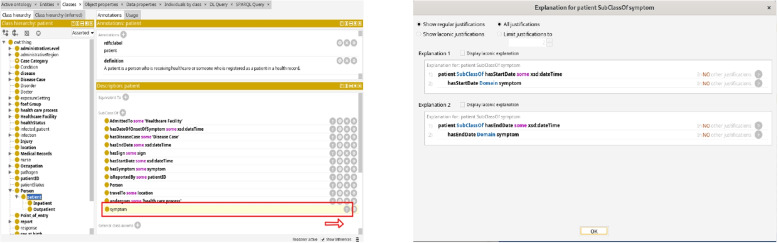
Ontology structure showing how patient is inferred to be a subclass of symptom through property restrictions and domain declarations

**Fig. 10:**
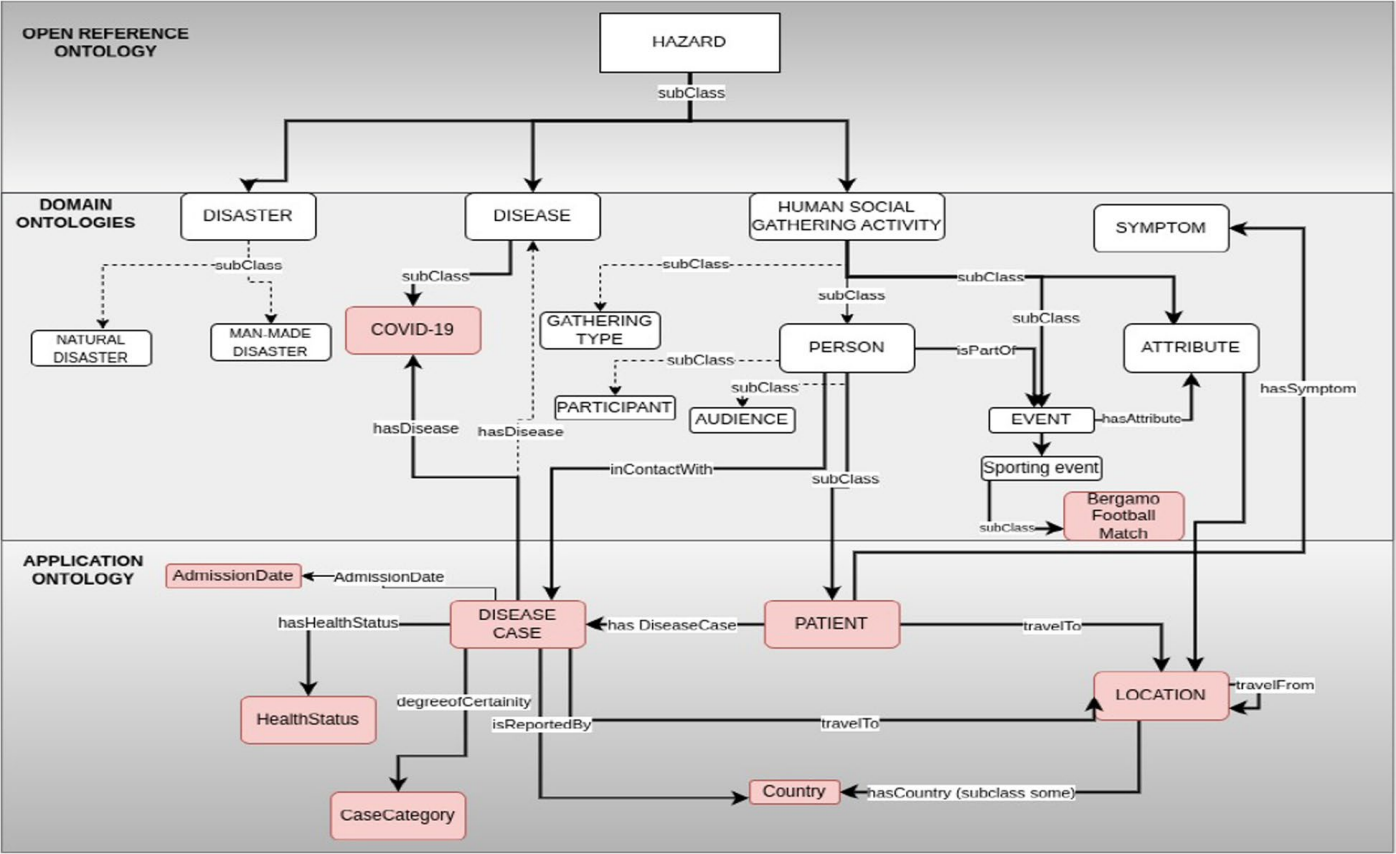
Integrating and inferring from the semantic framework

**Fig. 11:**
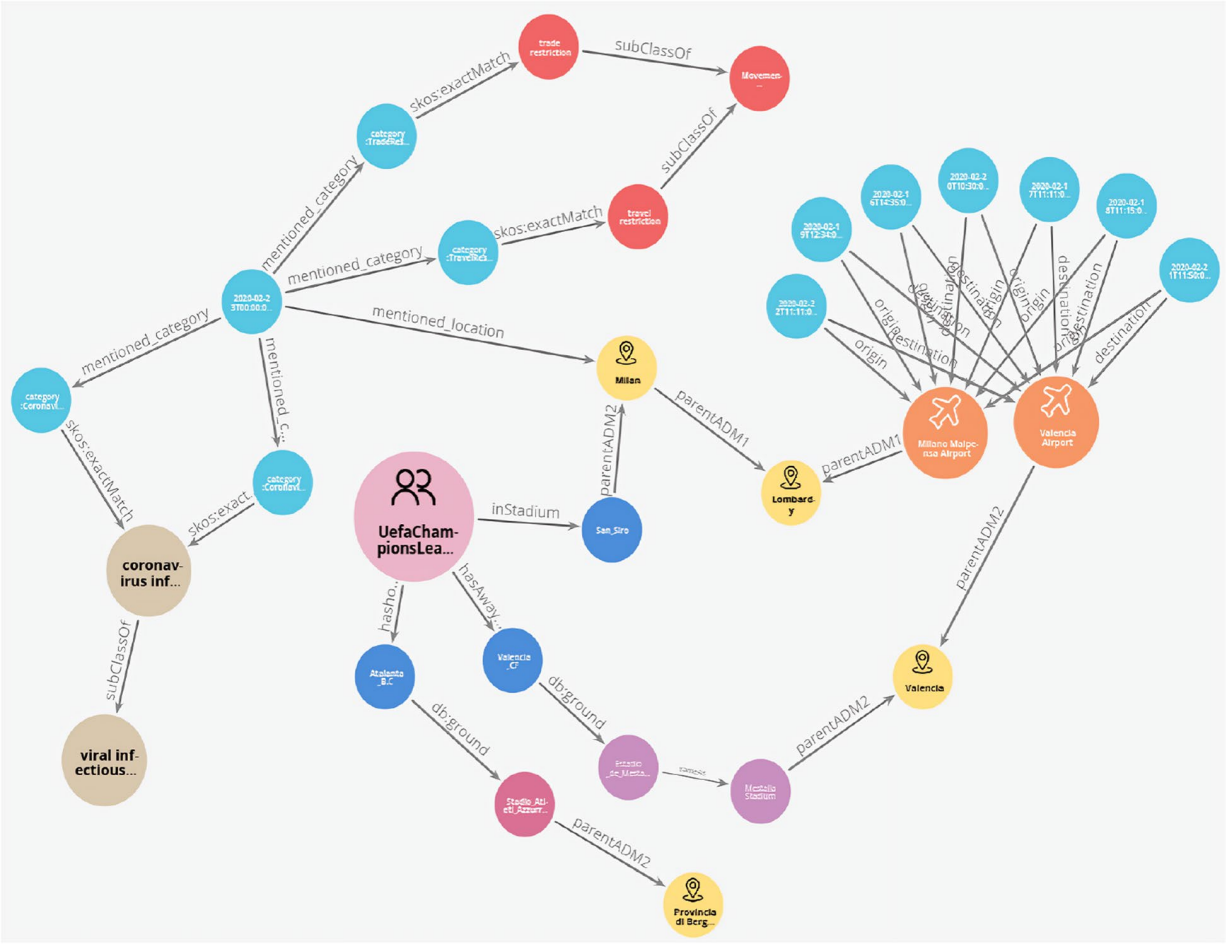
Property graph

Here, we present the key outcomes of our study:

1. **Semantic Enrichment of Structured and Unstructured Data:** The semantic framework and ontologies were instrumental in enriching structured and unstructured data, typically stored in databases, Excel spreadsheets, CSV files or documents. This process involved mapping data elements to ontology concepts and creating RDF triples following the subject-predicate-object format. The SCAIVIEW^7^ tool was utilized to automatically tagged ontology concepts within unstructured documents, particularly EIOS documents. These documents were subsequently transformed into RDF triples using Simple Knowledge Organization System (SKOS) rules, and the resulting millions of triples were stored within the triple store. The ontology concepts and their relationships were instrumental in creating these data graphs.
  The semantic framework supported semantic enrichment by providing structured representations and contextual information for entities and concepts identified within both structured and unstructured data sources. For example, when a document references terms such as the FIFA World Cup, UEFA Champions League, or English Premier League, these are mapped to corresponding concepts within the Mass Gathering ontology. This mapping provides contextual understanding and allows the system to interpret these references as formal event entities, rather than isolated textual mentions. These entities are further linked to additional contextual details—such as location, date, and participating teams—as specified within the ontology. Through this process, the raw data is semantically enriched with domain-specific knowledge, enhancing its clarity, interpretability, and potential for analysis.
2. **Integration and Inference within the Semantic Framework:** The integration of the defined ontologies from all the layers mentioned above creates a unified framework for public health knowledge representation and inference generation. The framework facilitates in data integration from different sources. An example of integration is demonstrated through a SPARQL query (Listing 1) that combines data from sport fixtures, stadiums, flights, airports, and Geonames. This shows how the framework enables querying across multiple datasets to extract relevant information. The results are shown in Fig. 6.
  The semantic framework also supports inference through its layered ontology architecture. By structuring ontologies across different layers, we enable reasoning that goes beyond explicitly stated facts. As shown in Figs. 7 and 8, we illustrate the difference in knowledge representation for an entity, *person1*, of type patient. Without inference, the system only identifies *person1* as a patient based on direct assertions. However, with inference enabled and ontologies connected, the framework can derive that *person1* is not only a patient but also a person involved in a mass gathering, exposed to potential hazards, and relevant to public health monitoring. Figure 9 further demonstrates the ontology structure, showing how patient is inferred to be associated with symptom through property restrictions and domain declarations. This highlights how semantic relationships and inference rules enhance the depth and utility of integrated health data.
  The application ontology based on the COVID line list dataset serves as a hub in our case, connecting to other ontologies and enhancing our contextual understanding of COVID-19-related information. Figure 10 provides a holistic view of the interconnected layers of ontologies and their role in facilitating inferencing through concept reuse. The bold lines in the figure depict the patient’s journey, illustrating the interconnectedness of the three layers. The classes like Disease case and Patient offer insights into patient travel history, exposure locations, health status, occupation, case categories, and more.
  Interestingly, the Person class in the COVID ontology is reused from the mass gathering ontology, where it has sub-classes like Participant. When utilized, the Patient class establishes links or merges with the mass gathering ontology through the ’Person’ class. The Hazard concept, originating from the open reference ontology, also connects with concepts such as Human social gathering activity, Disease, and Symptom from the same open reference ontology. These concepts expand further into the *mass gathering ontology, disease ontology, and symptom ontology* respectively, within the domain ontology layer. These domain-specific ontologies provide comprehensive information about gathering events, diseases like COVID-19, and disease symptoms.
3. **Creation of Linked Data:** The semantic framework forms the basis for creating linked data by enabling the integration of structured and unstructured sources into a unified representation. It provides a standardized way to model entities and relationships using domain ontologies, which helps in reducing ambiguity and ensuring consistency across datasets. This standardization facilitates more accurate and reliable querying. In addition, the framework allows queries to span across different sources, making it possible to trace meaningful paths between related entities, for instance, linking disease mentions in text with air travel data and mass gathering events—to support context-rich analysis in the public health domain. This is illustrated in Fig. 11.
4. **Application Ontology for COVID-19 Linelist Data:** We developed an application ontology to address the specific needs of the COVID-19 linelist data. This ontology meticulously represented the dataset’s columns and unique values. By converting the CSV dataset to RDF, we generated RDF triples that linked the line list data to related concepts, including geographic concepts from geonames, spatiotemporal concepts from the travel ontology, and disease-related concepts to DOID ontology which are all covered in the domain ontology layer and linked in the open reference ontology layer (Table 5).
5. **Knowledge Graph Utilization:** The knowledge graphs stored in the triple store can serve as a foundational resource for anomaly detection algorithms. These algorithms can leverage the rich semantic relationships encoded in the knowledge graphs to identify patterns, outliers, and anomalies within the data, along with other potential applications. As illustrated in Fig. 10, a person’s movements can be traced through the events they attended, while simultaneously identifying whether they were a patient or had contact with one. In our use case, we constructed a knowledge graph (Fig. 11) by integrating data from multiple sources, including the sports fixture dataset, flight records, EIOS documents, and the COVID-19 line list. The results demonstrate how we can establish connections between a disease mention in a news article, an ongoing event such as the Champions League, and corresponding flights from cities, highlighting the ability of our framework to unify contextual information across domains.

**Figure:**
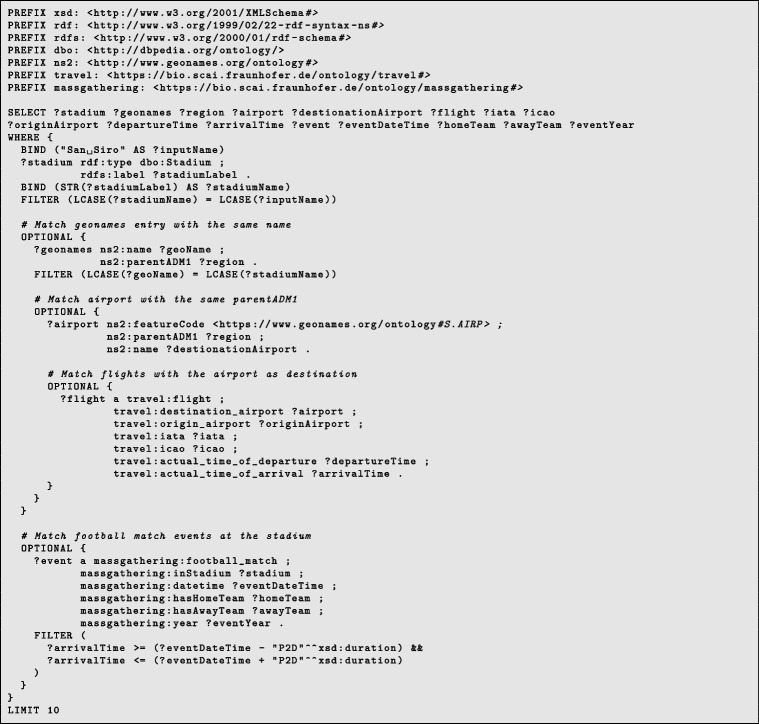
**Listing 1** SPARQL query illustrating semantic integration of heterogeneous data sources

**Table 5:** RDF dataset summary

Data source	Raw data size	RDF triple count
EIOS	4.2 million documents	1.5 billion triples
WHO COVID-19 Linelist	1516854 rows, 142 columns	112.3 million triples
FlightAware dataset	2335981 rows, 25 columns	14741854 triples
Sports fixtures	19763 rows, 7 columns	244571 triples
Influenza reports	400446 rows, 40 columns	39494858 triples

## Discussion

In pursuing enhanced pandemic preparedness and prevention, our approach extends beyond conventional disease and hospital information ontologies, as public health intelligence surpasses the traditional scope of disease and hospital surveillance. A more extensive set of information encompassing the origins, targets, and contextual elements becomes crucial to discern the spread of infections and institute preemptive measures. Utilizing multiple ontologies rather than a single overarching ontology can offer several advantages. One of the key benefits of employing multiple ontologies is reducing complexity by breaking down large ontologies into smaller, more manageable pieces. This modular approach can facilitate ontology development and maintenance, enabling focused updates and iterative improvements. Additionally, it allows different groups to develop ontologies that are tailored to their specific needs, promoting collaboration and customization within the domain.

The ontologies and semantic framework developed in this project are tailored to the specific use case and due to time constraints, it was not feasible to encompass all restrictions within a general structure. Consequently, certain concepts are situated within the ontology based on contextual considerations of where they should be defined. For instance, the classification of"Mass gathering"is designated under the hazard category in accordance with our particular use case. In reality, such categorization would only occur when specific conditions are met, indicating the transition of a mass gathering into a hazardous situation.

While employing a semantic framework in public health offers potential benefits, there are inherent limitations to consider. Firstly, the semantic web, which underpins this approach, is still evolving, requiring training for both data providers and users. Secondly, mapping application ontologies to domain ontologies is a time-consuming task requiring expertise from domain specialists and data providers. Lastly, managing different ontology versions over time can become cumbersome. To address these limitations, standardized methodologies for ontology development and meticulous data annotation are essential for establishing meaningful relationships and enabling effective analysis within knowledge graphs.

## Conclusion and future directions

In this paper, we present a semantic framework that uses semantic technologies to integrate and interpret data from various sources. We utilized this framework to establish a semantic web or ‘web-of-data’. This allows public health officers with a unified data resource to perform querying and deeper data exploration.

Our framework adopts a three-layered architecture that enables the reuse of domain ontologies, ensures consistent knowledge representation, and facilitates the understanding of the data structure. The three layers are (1) a reference ontology that defines the basic concepts and relations common to all domains, (2) domain ontologies that extend the core ontology with specific concepts and relations for each domain, and (3) an application ontology built specifically for the application that maps the columns of each dataset to the corresponding concepts and relations in the domain ontology. While the domain ontologies provide a deep understanding of individual areas, the upper-level reference ontology plays a crucial role in integrating these domains within the broader public health context, facilitating cross-domain connections and enhancing comprehensive insights.

This project aims to highlight the value of linked data in public health. Shared vocabularies provided by ontologies enable researchers to annotate concepts within disparate datasets, fostering semantic enrichment and seamless integration and analysis. Drawing upon domain-specific and reference ontologies, the annotated data becomes interoperable and reusable across different systems. This approach leverages RDF triples to represent data as knowledge graphs, offering public health officers a unified resource with crucial contextual information for informed decision-making. Compared to traditional relational databases, RDF facilitates flexible and intuitive data exploration. Additionally, leveraging graph technologies allows researchers to perform knowledge inference, enabling them to discover new or unexpected insights from the data. Ultimately, this project aims to elevate awareness and understanding of linked data within the public health field.

The current framework is limited in scope, as it was developed to address a specific use case; however, it establishes a foundation that can be further expanded to encompass additional public health scenarios. Although it incorporates most key topics relevant to public health, the level of detail remains constrained. For instance, the framework could be extended to cover specific events such as the West Nile virus outbreak or wastewater-related epidemics. In such cases, domain ontologies would be revised to include relevant concepts, and the Open Reference Ontology could be augmented by introducing new subclasses such as *biological hazard* and *chemical hazard* under the existing *Hazard* class to more accurately represent these specific threats.

## Data Availability

All ontologies are available at: https://ols.ewaa.scaiview.com/ontologies.

## Footnotes

1 https://data.who.int/about/data/whdh/xmart

2 https://fixturedownload.com/

3 https://www.ebi.ac.uk/ols4/

4 https://ontofox.hegroup.org/

5 https://jena.apache.org/documentation/fuseki2/

6 https://jena.apache.org/documentation/fuseki2/fuseki-configuration.html#possible-reasoners

7 https://www.scai.fraunhofer.de/en/business-research-areas/bioinformatics/products/scaiview.html
